# RETRACTION: Effect of Manual Lymphatic Drainage Combined With Vacuum Sealing Drainage on Axillary Web Syndrome Caused by Breast Cancer Surgery

**DOI:** 10.1111/iwj.70555

**Published:** 2025-04-18

## Abstract

**RETRACTION:** LiuJ. , ChenD. , and YinX. , “Effect of Manual Lymphatic Drainage Combined With Vacuum Sealing Drainage on Axillary Web Syndrome Caused by Breast Cancer Surgery,” International Wound Journal 20, no. 1 (2023): 183–190, 10.1111/iwj.13862.35778796 PMC9797928

The above article, published online on 01 July 2022, in Wiley Online Library (http://onlinelibrary.wiley.com/), has been retracted by agreement between the journal Editor in Chief, Professor Keith Harding; and John Wiley & Sons Ltd. Following an investigation by the publisher, all parties have concluded that this article was accepted solely on the basis of a compromised peer review process. In addition, further investigation by the publisher found inconsistencies in the listed inclusion criteria and participant sections and that the article did not include information on how the QOLS had been calculated. The investigation also found that some included references were irrelevant or not discoverable online. In view of the clear evidence of compromised peer review, the parties agreed that the paper must be retracted. The authors did not respond to our notice regarding the retraction.

**RETRACTION:** J. Liu , D. Chen , and X. Yin , “Effect of Manual Lymphatic Drainage Combined With Vacuum Sealing Drainage on Axillary Web Syndrome Caused by Breast Cancer Surgery,” International Wound Journal 20, no. 1 (2023): 183–190, 10.1111/iwj.13862.35778796 PMC9797928

The above article, published online on 01 July 2022, in Wiley Online Library (http://onlinelibrary.wiley.com/), has been retracted by agreement between the journal Editor in Chief, Professor Keith Harding; and John Wiley & Sons Ltd. Following an investigation by the publisher, all parties have concluded that this article was accepted solely on the basis of a compromised peer review process. In addition, further investigation by the publisher found inconsistencies in the listed inclusion criteria and participant sections and that the article did not include information on how the QOLS had been calculated. The investigation also found that some included references were irrelevant or not discoverable online. In view of the clear evidence of compromised peer review, the parties agreed that the paper must be retracted. The authors did not respond to our notice regarding the retraction.

